# The socioeconomic burden of facioscapulohumeral muscular dystrophy

**DOI:** 10.1007/s00415-021-10591-w

**Published:** 2021-05-27

**Authors:** Anna M. Blokhuis, Johanna C. W. Deenen, Nicol C. Voermans, Baziel G. M. van Engelen, Wietske Kievit, Jan T. Groothuis

**Affiliations:** 1 Department of Rehabilitation, Sint Maartenskliniek, Nijmegen, The Netherlands; 2 Donders Institute for Brain, Cognition and Behaviour, Department of Rehabilitation, Radboud university medical center, Nijmegen, The Netherlands; 3 Donders Institute for Brain, Cognition and Behaviour, Department of Neurology, Radboud university medical center, Nijmegen, The Netherlands; 4 Department for Health Evidence, Radboud Institute for Health Sciences, Radboud university medical center, Nijmegen, The Netherlands

**Keywords:** Muscle disease, Cost, Burden, Quality of life

## Abstract

**Background:**

Promising genetic therapies are being investigated in facioscapulohumeral muscular dystrophy (FSHD). However, the current cost of illness is largely unknown.

**Objective:**

This study aimed at determining the socioeconomic burden of FSHD.

**Methods:**

Adult patients with FSHD from the Dutch FSHD registry were invited to complete a questionnaire on medical consumption, work productivity and health-related quality of life (HR-QoL) using the EQ-5D-5L*.* Associated costs were calculated from a societal perspective. A generalized linear model was fitted to the data to investigate whether level of mobility was related to annual costs of illness.

**Results:**

172 patients with FSHD completed the questionnaire (response rate 65%)*.* The per-patient annual direct medical costs of FSHD were estimated at €12,077, direct non-medical costs at €9179 and indirect costs at €5066, adding up to a total cost of illness of €26,322 per patient per year. The direct costs of illness were €21,256, approximately five times higher than the mean per-capita health expenditures in the Netherlands. Major cost-driving factors were formal home care and informal care. A decreased level of mobility was associated with higher direct costs of illness. HR-QoL was significantly reduced in patients with FSHD with a median health utility value of 0.63.

**Conclusions:**

We show that FSHD is associated with substantial direct and indirect socioeconomic costs as well as a reduction in HR-QoL. These findings are important for health care decision makers and aids in allocation of research funds and evaluation of the cost-effectiveness of novel therapies.

## Introduction

Facioscapulohumeral muscular dystrophy (FSHD) is the third most common inherited neuromuscular disorder with an estimated prevalence of 12 per 100,000 in the Netherlands [1]. Symptom severity of the disease is heterogeneous, ranging from minimal clinical manifestations to severe muscle weakness and wheelchair dependency. Respiratory function is normal in most patients, but in 1–3% of cases nocturnal ventilatory support is required [2, 3]. Life expectancy in FSHD is (near) normal, hence disease duration is long compared to many other neuromuscular diseases.

The current medical management of FSHD is supportive and includes, among other interventions, physical therapy, pain reduction treatment, and monitoring of respiratory function [4]. However, several trials of novel pharmaceutical treatments of FSHD are currently executed [5, 6]. The recent discovery of an effective antisense oligonucleotide therapy in spinal muscular atrophy [7] gives rise to hope that RNA-based therapies could be effective also in patients with FSHD [8–10]. Indeed, at the time of writing, several genetic therapies aimed at repressing DUX4 and its downstream effects are being investigated as potential treatment options in FSHD [11]. The increasing pace of development of novel therapies also fueled the ongoing debate on the costs of such health technologies for society as a number of recent orphan drugs are extremely expensive. New expensive therapies might be cost-effective if there is much to gain in terms of cost savings and improvement in quality of life for a specific disease. Therefore, the aim of the present study was to investigate the socioeconomic burden of FSHD by assessing medical consumption, work productivity and health-related quality of life in patients with FSHD.

## Methods

### Study design

We used a cross-sectional study design. Patients with FSHD registered within the Dutch FSHD registry were invited to fill out a questionnaire in August 2018.

### Recruitment of participants and study procedures

Data collection took place between August and November 2018. We contacted patients with FSHD via the Dutch FSHD registry [12]. Participants who were 18 years or older and residing in the Netherlands were invited via email to fill out a questionnaire using the online data management system Castor EDC. Five weeks after the initial invitation, a reminder was sent.

The questionnaire consisted of four parts. The first part included questions regarding patient and disease characteristics (e.g. age, sex, education, current occupation, symptom severity, level of mobility). If participants had recently provided this information as part of the registry, these data were derived from the registry. The level of education was classified according to information from Statistics Netherlands and summarized as low, middle or high [13]. The second part of the questionnaire was an adapted version of the Medical Consumption Questionnaire from the institute for Medical Technology Assessment (iMTA-MCQ) [14], a generic instrument for measuring health care utilization. This part of the questionnaire included questions on hospital admissions, contacts with health care providers, medication use, ventilatory support, purchase of medical aids, investments in house adaptations and formal caregiving. A recall period of 3 months is used in the iMTA-MCQ, with the exception of adaptations and aids for which the recall period is 1 year. For the evaluation of informal care giving a selection of questions from the iMTA Valuation of Informal Care Questionnaire (iVICQ) was used [15]. The recall period for informal care in this questionnaire is 1 week. The third part of the questionnaire consisted of the Productivity Cost Questionnaire from the iMTA (iMTA-PCQ) which measures loss in productivity from both paid and unpaid work due to illness [16]. The iMTA-PCQ includes questions on whether participants have been absent from work due to illness. Patients are asked whether they have been less productive at work due to their illness in the past 4 weeks (so-called presenteeism) and whether they could perform less unpaid work due to illness in the past 4 weeks. Finally, the EQ-5D-5L was incorporated to assess patient health-related quality of life (HR-QoL) [17]. The EQ-5D-5L is a widely used instrument to measure health status and consists of two parts. The first part assesses health in five dimensions (mobility, self-care, usual activities, pain/discomfort and anxiety/depression) using five response levels. The descriptive profile derived from these questions can be used to calculate a health utility value. A health utility reflects how good or bad a health status is according to the preferences of the general population of a specific country with 1 indicating perfect health and 0 death. The second part of the EQ-5D-5L consists of a visual analogue scale (VAS) on which perceived health is rated from 0 (worst imaginable health) to 100 (best imaginable health).

### Patient informed consent and ethical approval

The questionnaires were incorporated in the Dutch FSHD registry after approval of the registry steering board, therefore, the informed consent for participation in the registry sufficed. Study ethical approval was granted by the Medical Ethical Committee of the Radboud university medical center (2018–4055).

### Cost assessment

Costs of illness were considered from a societal perspective, including direct medical costs, direct non-medical costs and indirect costs related to production losses in line with the Dutch guideline for economic evaluations in health care [18, 19].

## Direct medical costs

Direct medical costs included hospital admissions, physician visits, visits to other health care professionals, medication, formal home care and ventilation. To calculate costs of medical consumption we made use of Dutch health care reference prices [18], which were corrected for inflation using the iMTA costing tool to reflect 2018 values [20]. For dietician visits, home mechanical ventilation and out-patient rehabilitation treatment, no references prices were available. Therefore, we made an estimation of costs based on price information from three dietician practices (€32 per hour), information from the Centre for Home Mechanical Ventilation Utrecht (€8500 per year for non-invasive and €13,000 per year for invasive ventilation) and the price of two hours of out-patient rehabilitation treatment, respectively. Medication costs were calculated using medication prices from the Dutch National Health Care Institute [21]. A standard price for delivery costs of the pharmacy was included in the calculation [18].

## Direct non-medical costs

Direct non-medical costs included costs for house adaptations, aids and devices, informal care and travel costs. With regard to expenditures for medical aids and home adaptations, we used patient-reported data. In some cases, patients reported unrealistic low prices, likely because costs were (partly) covered by health or social insurances. In these cases we consulted the aid category provided by the Dutch National Health Care Institute [22]. If no information was available, we made an estimation based on expert opinion considering the lowest reasonable price. Hours of home care and informal caregiving were maximized at 112 h a week in total with the rationale that 16 h of care per day is the maximum, to allow 8 h of sleep. There are a number of methods available to value informal care, depending on the perspective of the evaluation [23]. In line with the national recommendations we valued informal care using the reference price from the guideline (based on the proxy good method; 2018 value €14.57) [18]. Where applicable, costs were annualized under the assumption that a similar amount of costs would be made in any given equal period of time.

## Indirect costs

Indirect costs included costs due to loss of productivity both in paid and voluntary work. Loss of productivity was calculated according to the friction cost method [24]. This method is based on the assumption that in case of prolonged absence, absentees will ultimately be replaced. The level of unemployment at a particular timepoint will determine the time span needed for an organization to find replacement (the friction period). From a societal perspective, the cost for productivity loss is limited to this friction period. For this study, the friction period was calculated (109 days) based on the number of vacant and filled jobs in 2018 as derived from Statistics Netherlands [25]. Loss of productivity of paid work was valued at the Dutch average wage rates and loss of unpaid work was valued at costs for household care according to the Dutch guideline [18].

As a sensitivity analysis and to enable comparison to other cost of illness studies, we also estimated costs due to productivity loss according to the Human Capital Approach. We valued loss of productivity due to temporary and permanent illness including sick leave, premature retirement and permanent disability but excluding valuation of presenteeism and unpaid work. In the Netherlands, if an employee becomes unable to work, the employer will pay at least 70% of income up to a maximum of 2 years. After 2 years of illness, it will be considered whether the employee is eligible for an invalidity pension, referred to as permanent disability in this study. The cost of temporary illness was calculated using Dutch average wage rates adjusted by the actual working hours of the patients concerned. Costs of permanent illness were calculated using mean annual Dutch average wage rates and only if FSHD was the reason for early retirement or permanent disability.

## Health-related quality of life (HR-QoL)

Health utility values were calculated using the Dutch tariff for the EQ-5D-5L [26]. Intangible costs were estimated by assigning a monetary value to the difference between the EQ-5D-5L derived health utility and the age-specific mean health utility in the Dutch population for each patient. A value of €50,000 per quality-adjusted life year (QALY) was used in the analysis. Costs are presented in euros (2018 values).

### Statistical analysis

Statistical analyses were performed using IBM SPSS Statistics 25. As cost data often show a right-skewed distribution, mean costs and bootstrap 95% confidence intervals were estimated by applying the bias corrected and accelerated technique implemented in IBM SPPS Statistics. Mean and standard deviation (SD) were reported for normally distributed data, median and interquartile range (IQR) in case of skewed data, because the mean is highly sensitive for outliers. Differences in total costs between subgroups were analyzed using the Mann–Whitney U test and Kruskal–Wallis test. A generalized linear model with an identity link function and gamma distribution was fitted to the data to investigate whether the mean per-patient annual costs of illness varied among the three mobility classes (walking without mobility aid, walking with mobility aid, unable to walk) and to predict costs for these groups [27]. We focused on mobility as a determinant of costs as it may be regarded as a proxy for disease severity. Before we performed the generalized linear model, we selected age, comorbidity, educational level and sex as potential confounders. Then we explored whether these potential confounders differed across the three levels of mobility which was the case for age, educational level and comorbidity but not sex. As well, when added as predictors in a regression model, these three factors altered the standard coefficient beta of the factor mobility. Therefore, to control for confounding effects, the generalized linear model was adjusted for age, educational level and comorbidity. The significance level was set to 5%.

## Results

### Participants

A total of 268 patients with FSHD were invited to fill out the questionnaire. Of those, 182 patients responded and 175 completed the full questionnaire, resulting in a response rate of 65% (responded and completed). Three patients were not officially diagnosed by a clinician and were excluded from the analysis. Of all patients with FSHD, 31% were diagnosed with FSHD type 1, 4% with FSHD type 2, 1% with FSHD type 1 and 2 and in 64% of cases the type of FSHD was unknown or not mentioned. Patient characteristics are presented in Table 1. The participants had a median age of 56 years (range 18–80). Median age at symptom onset was 20 years (range 0–70), and mean disease duration at the time of filling out the questionnaire was 30 years (range 0–75).

**Table 1:** Characteristics of FSHD patients included in this study (*n* = 172)

Age (years)	
Median (IQR)	56 (45–66)
Range	18–80
Sex, female	94 (55)
Level of education	
Low	37 (22)
Middle	65 (38)
High	70 (41)
Current main occupation (single selection)	
Student	2 (1)
Paid employment	58 (34)
Self-employed	11 (6)
Housework	8 (5)
Voluntary work	5 (3)
Unemployed	1 (1)
Permanent disability	42 (24)
Premature retirement	4 (2)
Retired	39 (23)
Other	2 (1)
Muscle weakness (patient-reported)	
Facial weakness	102 (59)
Shoulder weakness	161 (94)
Pelvic and proximal leg weakness	140 (81)
Axial muscle weakness	141 (82)
Age of onset (years)	
Median (IQR)	20 (12–40)
Range	0–70
Disease duration	
Mean (SD)	30 (16)
Range	0–75
Level of mobility	
Without mobility aid	79 (46)
With mobility aid	73 (42)
Non-walker	20 (12)
Wheelchair use	
All day	20 (12)
Part of the day	27 (16)
No wheelchair	125 (73)

Data presented as *n* (proportion %) if not otherwise specified

*IQR* interquartile range, *SD* standard deviation

### Medical consumption

Per-patient annual medical consumption is depicted in Table 2. Patients with FSHD made use of a variety of health care resources. In the preceding 3 months, 53% of patients visited a general practitioner, 26% consulted a rehabilitation physician and 19% visited a neurologist. Furthermore, 58% of patients consulted a physical therapist, 20% an occupational therapist and 10% a psychologist in the preceding 3 months. Of all patients, 42% received informal care and 19% home care. Mean hours of informal care giving was 22 h per week (SD 18 h, range 1–56 h per week). Moreover, 36% of participants purchased aids or devices and 21% made adaptations to the house in the past year. Hospital admissions were rare in the preceding 3 months (3%).

**Table 2:** Utilization of medical resources by FSHD patients in the preceding 3 months (*n* = 172)

Hospital clinical care	
Emergency ward admission(s)	7 (4)
Hospital admission(s)	5 (3)
Median duration hospital stay in days (IQR)	5 (2–7)
Hospital day admission(s)	9 (5)
Ambulance transport	2 (1)
Non-hospital institutional care (e.g. nursing home or rehabilitation center)	
Non-hospital clinical care	1 (1)
Non hospital day admissions	9 (5)
Visits to physicians	
General practitioner	81 (47)
Specialist physician	78 (45)
Rehabilitation doctor	45 (26)
Neurologist	33 (19)
Cardiologist	10 (6)
Internist	9 (5)
Company doctor	13 (8)
Visits to other health care professionals	
Physiotherapist	100 (58)
Occupational therapist	34 (20)
Speech-language therapist	9 (5)
Psychologist	17 (10)
Dietician	14 (8)
Social worker	8 (5)
Ventilatory support	
Non-invasive, < 24 h a day	6 (3)
Non-invasive, 24 h a day	0 (0)
Invasive, < 24 h a day	1 (1)
Invasive, 24 h a day	0 (0)
Home care	33 (19)
Informal care giving	72 (42)
Adaptations, aids and devices^a^	
Home adaptations	36 (21)
Aids and devices	62 (36)

Data presented as *n* (proportion %) if not otherwise specified

*IQR* interquartile range

^a^ Recall period for adaptations aids and devices is 1 year, recall period for all other services is 3 months

### Productivity in paid and voluntary work

Of all patients, 42% had a paid job, 23% was retired and 24% was permanently disabled. Of all participants with a permanent disability, 93% mentioned FSHD as the cause of their disability. Productivity losses due to absence from work in the past 4 weeks was reported by 12% of paid workers. Of those who reported absenteeism, 67% had been absent for more than 4 weeks with a median number of 152 days (~ 22 weeks) absent from work at the moment of filling out the questionnaire. Importantly, almost half of all paid workers (48%) reported that their health condition prevented them from being fully productive at work, also known as presenteeism. Considering unpaid work, such as household work and volunteer work, 28% of patients with FSHD noted that they could perform less unpaid work as a result of health problems.

### Quality of life

**Fig. 1:**
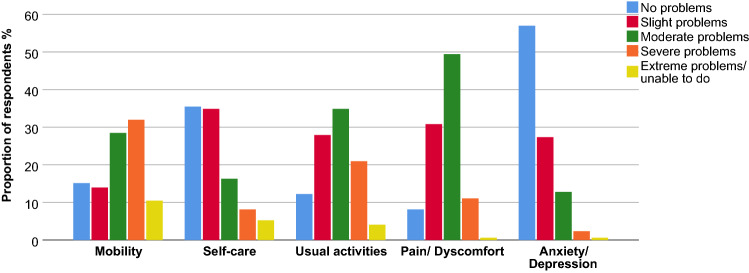
Proportion of responses by level of severity for EQ-5D-5L dimensions

The results of the EQ-5D-5L are summarized in Fig. 1 and Table 3. The majority of patients with FSHD had problems with mobility, self-care and daily activities. Pain or discomfort was very common with only 8% of respondents having no problems in this domain. Slight to extreme problems with regard to anxiety or depression were reported in 43% of cases. Using the Dutch value set that reflects the preferences of the general population [26], we calculated a median health utility value of 0.63 (IQR 0.40–0.75) for the FSHD group. The median difference between the EQ-5D-5L derived health utility and the age-specific mean health utility in the Dutch population for each patient was 0.25 (IQR 0.11–0.45). Health utility was lower in patients walking with a mobility aid compared to patients walking without a mobility aid (p < 0.001) and lower in patients unable to walk compared to patients walking with a mobility aid (p = 0.02) (Table 3).

**Table 3:** Quality of life evaluation of FSHD patients using the EQ-5D-5L (*n* = 172)

Utility value total group	
Median (IQR)	0.63 (0.40–0.75)
Mean (SD; 95% CI)	0.57 (0.26; 0.53–0.61)
Range	−0.22 to 1.00
Utility value by level of mobility,	
median (IQR)	
Walking without mobility aid	0.74 (0.64–0.82)
Walking with mobility aid	0.56 (0.30–0.65)
Unable to walk	0.38 (0.30–0.43)
Normative Dutch population (mean ± SD)[26]	0.87 ± 0.17
EQ-VAS median (IQR)	66 (50–75)

*IQR* interquartile range, *SD* standard deviation, *95% CI* 95% confidence interval

### Costs of illness

**Fig. 2:**
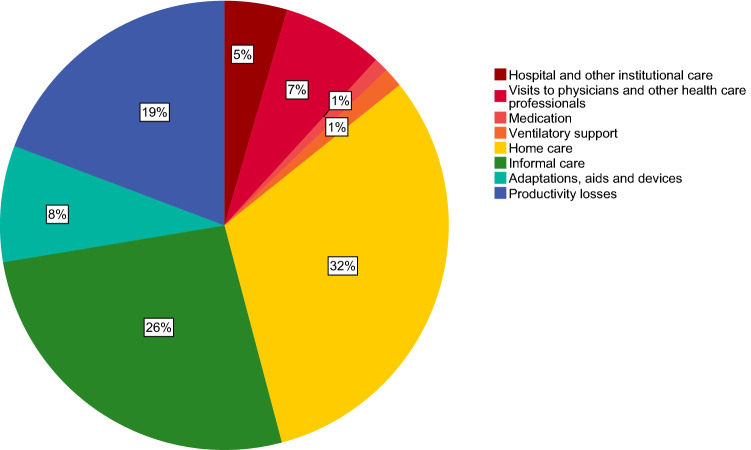
Distribution of cost components. Hospital care and other institutional care includes ambulance transport, tests and assessments and clinical and day care in rehabilitation center or nursing home. Travel costs are included in informal care

Per-patient annual costs of illness are presented in Table 4. The distribution of cost components is presented in Fig. 2. The total mean sum of direct medical costs was calculated at €12,077 per patient per year. Total direct non-medical costs were estimated at €9179 per patient per year. Of these costs, out-of-pocket payments by patients related to their health were €1306 on average per patient per year and included co-payments, non-reimbursed payments for aids and devices, adaptations and domestic help. Indirect costs (productivity losses in paid and unpaid work) accounted for €5066 per patient per year according to the Friction Cost Analysis. Valuation of temporary and permanent illness according to the Human Capital Approach estimated indirect costs at €12,921 per patient per year compared to €722 for absenteeism according to the Friction Cost Analysis.

**Table 4:** Per-patient annual costs of FSHD in euros (2018 values)

	Mean in € (95% CI)
Hospital clinical care	524 (218–936)
Emergency ward admissions	56 (19–105)
Hospital admissions	322 (69–656)
Hospital day admissions	145 (53–259)
Ambulance transport	37 (0–75)
Non-hospital institutional care (e.g. nursing home or rehabilitation center)	604 (118–1367)
Non-hospital clinical care	374 (0–749)
Non hospital day admissions	229 (89–407)
Visits to physicians	560 (449–678)
General practitioner	116 (91–145)
Specialist physician	395 (306–489)
Company doctor	49 (27–75)
Visits to other health care professionals	1362 (1130–1585)
Physiotherapist	1085 (899–1291)
Occupational therapist	63 (41–88)
Speech-language therapist	15 (4–30)
Psychologist	145 (75–227)
Dietician	14 (7–22)
Social worker	40 (11–76)
Medication	273 (161–423)
Tests and assessments	23 (5–48)
Ventilatory support	372 (148–645)
Home care	8322 (4702–13,029)
**Total direct medical costs**	**12,077 (7287–17,447)**
Informal care giving	6960 (5233–8886)
Adaptations, aids and devices	2205 (1442–3157)
Adaptations	838 (421–1287)
Aids and devices	1367 (815–2108)
Transport	14 (8–23)
**Total direct non-medical costs**	**9179 (7122–11,551)**
**Total indirect costs (productivity losses)**	**5066 (3521–6787)**
Absenteeism paid work	722 (259–1273)
Presenteeism paid work	1424 (884–2123)
Unpaid work	2920 (1786–4246)
**Total cost of illness**	**26,322 (20,355–33,787)**
Intangible costs (loss of quality of life)	14,528 (12,671–16,475)
**Total burden of illness**	**40,850 (33,722–49,411)**

*95% CI* 95% bootstrap confidence interval, *IQR* interquartile range

**Fig. 3:**
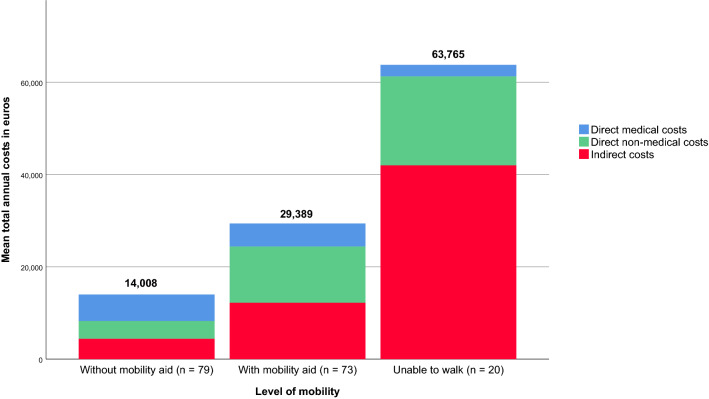
Mean per-patient annual costs of illness by level of mobility

Total annual costs of illness added up to €26,322 per patient per year. When assigning a monetary value to the loss in patients’ quality of life, the total cost would increase with €14,528 per patient per year. Figure 3 presents cost estimates stratified by level of mobility and shows that cost of illness increase with decreasing mobility. By means of the generalized linear model we estimated that total cost were €12,185 (*p* = 0.003) higher for patients walking with a mobility aid compared to patients walking without a mobility aid, adjusted for age, educational level and comorbidity. Being unable to walk increased total cost of illness with €36,382 (*p* = 0.001) compared to patients walking with a mobility aid, adjusted for age, educational level and comorbidity.

## Discussion

We studied the socioeconomic burden of FSHD in a large group of patients within the Dutch FSHD registry and estimated the mean per patient annual total cost of illness at €26,322. Direct medical and non-medical costs of illness were estimated at €21,256 per patient per year, approximately five times higher than the mean per-capita health expenditures in the Netherlands (€4480 in 2018) [28]. Major cost-driving factors were formal home care and informal care. HR-QoL was significantly reduced in patients with FSHD compared to the general Dutch population. The total socioeconomic burden of illness, including a monetary value of the loss in patient’s quality of life, was estimated at €40,850 per patient per year. So, FSHD is associated with considerable societal costs.

To our knowledge, there is only one earlier study that investigated the socioeconomic burden of FSHD [29]. This German study was much smaller than our study (*n* = 20 patients with FSHD), but calculated total costs of illness were remarkably similar (€26,240; 2009 value). However, a different approach in cost estimation was used as informal care was valued at the full wage rate and productivity loss was estimated according to the Human Capital Approach. The Human Capital Approach values all absenteeism until retirement as production loss, while the friction cost method, as used in the current study, values production loss for the time until replacement of the worker, generally resulting in a substantial lower estimation of costs as was also found in this study [30].

Compared to other neuromuscular diseases, total estimated costs of illness in FSHD were higher than earlier reported costs in myasthenia gravis (€14,950; 2009 value [29]; wide range of values in a recent systematic review [31]) and Charcot–Marie–Tooth neuropathy (€17,427; 2015 value) [32], similar to myotonic dystrophy (€27,497; 2010 value) [33], but generally lower than in ALS (review: range €12,313-€62,597; 2010 value; recent study: €78,256; 2018 value) [34, 35], spinal muscular atrophy (€70,566; 2013 value) [36] and Duchenne muscular dystrophy (study in four countries, range €34,723–62,476; 2012 values) [37]. However, a direct comparison between cost of illness studies must be interpreted with caution due to differences in the health care system per country and different approaches in cost estimation per study. Factors that have a particularly large influence on the outcome of economic evaluations are the valuation of informal care and productivity loss. Moreover, while the annual per patient costs might be higher in other neuromuscular diseases, survival is significantly shorter in these diseases and thus cumulative costs may be greater in FSHD. With approximately 2000 individuals being affected in the Netherlands [1], the total national cost of FSHD is estimated at €53 million per year in the Netherlands.

We showed that decreased mobility, which could be regarded as a proxy for disease severity, was associated with higher direct costs of illness. Costs for productivity losses showed an inverse relationship with mobility, with higher losses in the more mobile groups. This can be explained by the fact that more severely affected patients are less likely to take part in paid or voluntary work. Decreased mobility was also associated with a lower HR-QoL. Therefore, if a new therapy halts disease progression and prevents further immobilization, this would likely lower societal costs and improve HR-QoL. Even an expensive drug could then be cost-effective. Whether new therapies are truly cost-effective will depend on the effectiveness of the therapy concerned in terms of improvement in health condition and quality of life and needs to be evaluated in a cost-effectiveness study.

Patients with FSHD experience impairments in daily life and work and need support to function as optimal as possible. This is underlined by the results of this study showing that 58% of patients received physical therapy, 19% received home care and as much as 42% of patients received informal care. The high level of informal care also stresses the importance of adequate support to informal caregivers. Moreover, participation in the work force was relatively low (42%), while the level of permanent disability was high (24%) and almost half of all paid workers (48%) reported a health-related diminished productivity at work (presenteeism). An earlier Dutch FSHD study also found high levels of disability, although general employment level was higher, probably due to the fact that the study population was younger (range 22–61 years) [38, 39]. Importantly, about one-third of disabled people in the Netherlands who are not working would want to work and one-third of the ones working have a need for more adaptations at work [40]. This suggests that increased attention for work participation and adequate support while working is warranted in FSHD.

Although our study focused on the societal perspective, we did include a number of questions regarding costs from the patient perspective. The majority of costs were covered by the government or insurance companies, still on average €1306 was spent on out-of-pocket payments per patient per year. Furthermore, the high level of informal care giving and low level of labor force participation compared to the general age-matched Dutch population [41] will likely result in a substantial loss of income for the households concerned. This means that the burden for patients and their families could be even higher than expressed in this study.

Patients with FSHD had a significantly impaired HR-QoL with a median health utility of 0.63. In line with earlier reports on quality of life in FSHD [42–44], chronic pain was common as well as impairments in mobility and daily activities. Earlier studies have reported a negative relationship between pain and quality of life in FSHD [42, 43]. Unfortunately, fatigue is not part of the EQ-5D-5L, but it is known that fatigue is common in FSHD and is associated with a reduced quality of life [45]. Compared to other studies that used the EQ-5D-5L to evaluate HR-QoL in neuromuscular diseases, patients with FSHD had a higher quality of life than patients with ALS [35, 44], SMA [46, 47] or Duchenne muscular dystrophy [37], but lower than, e.g. in patients with myasthenia gravis [44].

Our study has several limitations. The inclusion of patients through the patient registry implies a selection bias. Although the health care workers involved in FSHD care at the Radboud university medical center repeatedly encourage all patients with FSHD in the Netherlands to take part in the Dutch FSHD registry, eventual registration is patient-initiated and the registry might, therefore, not be representative of the entire FSHD patient population. It is also possible that patients who do not take part in the work force have more spare time and are more likely to participate. On the other hand, the most severely impaired patients might not have been able to participate in the study. We noticed that educational level was relatively high in the study population compared to the general Dutch population. As well, mean age was relatively high in our study cohort and this might have resulted in an overestimation of costs. Unfortunately, genetic information on fragment size is not available within the registry at the moment, which is another limitation of the study. Furthermore, because we asked patients to retrospectively report on medical consumption, this likely resulted in a recall bias and underestimation of costs. Finally, health care systems and specifically unit prices vary between countries which limits the generalizability of our results to other countries. It is, therefore, recommended to specifically compare volumes of care as presented in this paper to another situation or country and estimate total costs using prices specific for that situation.

Our results highlight the substantial burden of FSHD for patients and society. As societal costs of FSHD are considerable and HR-QoL is reduced compared to the general population, there is much to gain in terms of cost savings and improvement in quality of life. This could give guidance for developers of new treatment modalities, it could serve as important information for health care decision makers and aids in the evaluation of new treatments for FSHD patients.

## Data Availability

Anonymized data will be shared by request from any qualified investigator in consultation with the FSHD registry.
